# Supplementary material for: MetaRibo-Seq measures translation in microbiomes
Source: Nat Commun. 2020 Jun 29;11:3268. doi: 10.1038/s41467-020-17081-z (PMC7324362; doi:10.1038/s41467-020-17081-z)
Supplement: Supplementary file 10 — Supplementary Data 7 [file 41467_2020_17081_MOESM10_ESM.zip › File2/Confidence_VeryHigh_Taxonomy/33903_out.krona.html]

Javascript must be enabled to view this page.

members
magnitude
magnitudeUnassigned
count
unassigned
taxon
rank

33903\_out

20

2
20
superkingdom

phylum
20
1239

186801
20
class

186802
20
order

186803
family
2

species
1

SRS062654\_contig\_number\_15002
39491

genus
1
189330

88431

SRS893270\_contig\_number\_contig-100\_6237.108053
species
1

31979
18
family

1485
genus
17

1
species

SRS024331\_contig\_number\_33086
2292308

16
species

SRS014923\_contig\_number\_44936SRS016989\_contig\_number\_contig-100\_15330.51852SRS017521\_contig\_number\_39968SRS022609\_contig\_number\_contig-100\_1.273481SRS024435\_contig\_number\_33969SRS052027\_contig\_number\_19532SRS056259\_contig\_number\_31332SRS075821\_contig\_number\_23856SRS098571\_contig\_number\_41957SRS098644\_contig\_number\_contig-100\_1092.275914SRS140492\_contig\_number\_32019SRS147919\_contig\_number\_14607SRS149181\_contig\_number\_contig-100\_4.185283SRS893295\_contig\_number\_10299SRS893300\_contig\_number\_5308SRS893383\_contig\_number\_26380
411486

2305245

SRS019693\_contig\_number\_contig-100\_28923.28924
1
species
